# Effects of PDE3 Inhibitor Olprinone on the Respiratory Parameters, Inflammation, and Apoptosis in an Experimental Model of Acute Respiratory Distress Syndrome

**DOI:** 10.3390/ijms21093382

**Published:** 2020-05-11

**Authors:** Petra Kosutova, Pavol Mikolka, Sona Balentova, Marian Adamkov, Andrea Calkovska, Daniela Mokra

**Affiliations:** 1 Biomedical Center Martin and Department of Physiology, Jessenius Faculty of Medicine in Martin, Comenius University in Bratislava, Martin 036 01, Slovakia; petra.kosutova@uniba.sk (P.K.); pavol.mikolka@uniba.sk (P.M.); andrea.calkovska@uniba.sk (A.C.); 2 Department of Histology and Embryology, Jessenius Faculty of Medicine in Martin, Comenius University in Bratislava, Martin 036 01, Slovakia; sona.balentova@uniba.sk (S.B.); marian.adamkov@uniba.sk (M.A.)

**Keywords:** olprinone, lung injury, inflammation, apoptosis, oxidative stress, PDE3

## Abstract

This study aimed to investigate whether a selective phosphodiesterase-3 (PDE3) inhibitor olprinone can positively influence the inflammation, apoptosis, and respiratory parameters in animals with acute respiratory distress syndrome (ARDS) model induced by repetitive saline lung lavage. Adult rabbits were divided into 3 groups: ARDS without therapy (ARDS), ARDS treated with olprinone *i.v.* (1 mg/kg; ARDS/PDE3), and healthy ventilated controls (Control), and were oxygen-ventilated for the following 4 h. Dynamic lung–thorax compliance (Cdyn), mean airway pressure (MAP), arterial oxygen saturation (SaO_2_), alveolar-arterial gradient (AAG), ratio between partial pressure of oxygen in arterial blood to a fraction of inspired oxygen (PaO_2_/FiO_2_), oxygenation index (OI), and ventilation efficiency index (VEI) were evaluated every hour. *Post mortem*, inflammatory and oxidative markers (interleukin (IL)-6, IL-1β, a receptor for advanced glycation end products (RAGE), IL-10, total antioxidant capacity (TAC), 3-nitrotyrosine (3NT), and malondialdehyde (MDA) and apoptosis (apoptotic index and caspase-3) were assessed in the lung tissue. Treatment with olprinone reduced the release of inflammatory mediators and markers of oxidative damage decreased apoptosis of epithelial cells and improved respiratory parameters. The results indicate a future potential of PDE3 inhibitors also in the therapy of ARDS.

## 1. Introduction

Acute respiratory distress syndrome (ARDS) is a common clinical disease that involves widespread inflammation in lung tissue, which is the first step in the development of lung dysfunction [1]. ARDS is characterized by acute onset (occurs within 1 week of the precipitating event) and the development of diffuse alveolar damage (DAD), which results in severe hypoxemia (PaO_2_/FiO_2_ ≤ 300 mmHg) and bilateral pulmonary infiltrates on chest radiography [2]. Bacterial and/or viral pneumonia are the most common causes of ARDS [3], although non-pulmonary sepsis, aspiration of gastric contents, trauma, blood transfusions, and pancreatitis also represent risk factors for the development of ARDS. In humans, the cardinal pathophysiologic features of ARDS include dysregulated inflammation and alveolar-capillary barrier disruption [4]. These are represented histologically by (a) neutrophilic alveolitis, (b) deposition of hyaline membranes in the alveoli, and (c) formation of microvascular thrombi, indicating activation of the coagulation cascade and endothelial damage [5]. Furthermore, increasing evidence has shown the interaction between inflammation and oxidative stress, which plays an important role in the pathogenesis of ARDS [6]. Oxidative stress, which is caused by a large concentration of reactive oxygen species (ROS), results in oxidative damage. However, anti-oxidant enzymes, such as catalase (CAT) or superoxide dismutase (SOD) can ameliorate this adverse condition [7]. These findings suggest that targeting the inflammation and/or oxidative stress could be a potential strategy to improve ARDS.

Clinical data analysis and animal studies have shown that excessive accumulation of neutrophils drives the development of ARDS [8,9]. Activated neutrophils can be effectively eliminated from the inflamed tissue by apoptosis [10]. Many mediators, such as tumor necrosis factor (TNF), interleukin (IL)-8, or IL-1 can suppress apoptosis of neutrophils, leading to their prolonged survival [11]. The delay of neutrophil apoptosis allows a large number of activated neutrophils to accumulate in the lung microvasculature and interstitium and persist for a long time. These neutrophils release several toxic factors, such as ROS, proteinases, and neutrophil extracellular traps, which can disrupt the endothelial–epithelial barrier [12]. The fate of neutrophils is one of the significant factors determining negative or positive consequences in the lung tissue while neutrophil apoptosis is thought to be an important control point in the resolution of inflammation.

Phosphodiesterases (PDE) are a family of enzymes that catalyze the inactivation of nucleotides cyclic adenosine monophosphate (cAMP) and cyclic guanosine monophosphate (cGMP) into 5′-AMP and 5′-GMP. Thus, PDE controls the concentration of these nucleotides and activate them to act on the intracellular signaling cascades [13].

Phosphodiesterase phosphorylation favors (a) the transcription of cAMP response element-binding protein (CREB) and activating transcription factor-1 (ATF-1), which induce the synthesis of anti-inflammatory cytokines and regulate the expression of genes related to cell growth and survival [14]. 

Indirect inhibition of transcription factor, the nuclear factor-κB (NF-κB), through the block by tyrosine kinase enzymes like mitogen-activated protein kinase (MAPK) [15]. Nuclear factor-κB is an indispensable protein complex in the activation of innate and acquired immunity, a response to cell stress, inflammation, and B-lymphocyte maturation [16]. 

Intracellular signaling through protein kinase A (PKA) regulates cell maturation and favors the synthesis of anti-inflammatory signals that inhibit the production of inflammatory mediators. Low cAMP concentrations favor inflammation due to the increase in IL-8, IL-12, IL-17, IL-22, IL-23, TNFα, γ-interferon, and chemokine C–X–C motif ligand 9 (CXCL9) and CXC10, and when the concentration increases, an anti-inflammatory response from anti-inflammatory cytokines are induced by the production of IL-6 and IL-10 [17].

Phosphodiesterase-3 (PDE3) is primarily expressed in the heart, lung, platelets, and white blood cells [18,19]. Inhibition of PDE3 showed anti-inflammatory [20], antioxidant [21], and anti-apoptotic actions in various cells and animal models [22]. In our previous experiments where the acute lung injury was induced by intratracheal meconium, intravenous olprinone significantly decreased the formation of oxidation markers in the lung, reduced lung edema, and prevented a decrease in total antioxidant status in the lung and plasma [23]. 

This study aimed to investigate whether PDE3 inhibitor olprinone can positively influence the inflammation, apoptosis, and respiratory parameters also in another model of ARDS induced by repetitive saline lung lavage.

## 2. Results

The body weights of animals and initial values of the parameters before induction of the ARDS model were comparable between the groups (all *p* > 0.05).

### 2.1. Cell Counts in the Bronchoalveolar Lavage Fluid (BALF)

In our study, saline lavage significantly increased the total number of cells in the bronchoalveolar lavage fluid (BALF) compared with the control group (ARDS vs. Control *p* < 0.01; Table 1), while olprinone partially prevented an increase in the total cells in BALF compared to ARDS group (ARDS/PDE3 vs. ARDS *p* < 0.05). 

Differential analysis of cell types in BALF showed an increase in macrophages, neutrophils, and eosinophils counts, with a prominent increase in neutrophils in the group of rabbits exposed to saline lavage (ARDS group) compared to healthy ventilated animals (Control group). Olprinone prevented the increases in all types of cells, particularly of neutrophils, compared with the ARDS group (Table 1).

### 2.2. Markers of Inflammation

**Figure 1:**
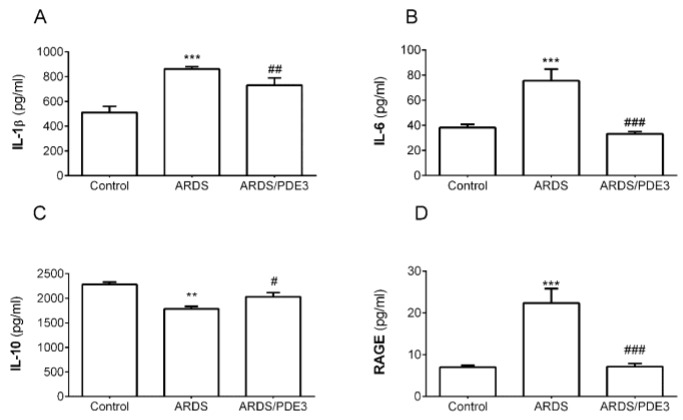
Levels of inflammatory cytokines (**A**) IL-1β, (**B**) IL-6, (**C**) IL-10, and (**D**) receptor for advanced glycation end products (RAGE) (all in pg/mL) in the lung tissue of healthy ventilated and non-treated animals (Control group), in non-treated animals with ARDS (ARDS group) and in animals with ARDS treated with olprinone (ARDS/PDE3 group) after the 4h therapy. Statistical comparisons: for ARDS vs. Control ** *p* < 0.01, *** *p* < 0.001; for ARDS/PDE3 vs. ARDS ^#^ *p* < 0.05, ^##^ *p* < 0.01, ^###^ *p* < 0.001. Data are presented as means ± SEM.

Lung lavage led to serious changes in all observed markers in the lung tissue. Pro-inflammatory cytokines IL-6 and IL-1β (both *p* < 0.001) and marker of lung epithelial cell injury RAGE (*p* < 0.001) increased and anti-inflammatory cytokine IL-10 (*p* < 0.01) significantly decreased in the saline-lavaged and untreated animals compared to controls (ARDS vs. Control). Olprinone therapy (Th) significantly reduced levels of IL-6 and RAGE (ARDS/PDE3 vs. ARDS, both *p* < 0.001), decreased IL-1β (ARDS/PDE3 vs. ARDS, *p* < 0.01), and increased IL-10 (ARDS/PDE3 vs. ARDS, *p* < 0.05) (Figure 1). 

### 2.3. Markers of Oxidative Damage

**Figure 2:**
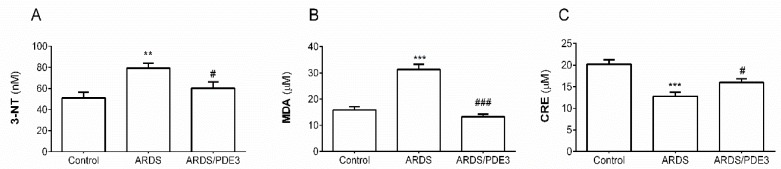
Levels of a marker of (**A**) oxidative modifications of proteins (expressed in nanomole concentration of 3-nitrotyrosine, 3NT), (**B**) a marker of lipid oxidation (thiobarbituric acid-reactive substances, TBARS, expressed in micromole concentration of malondialdehyde), and (**C**) total antioxidant capacity (TAC, expressed in micromole concentration of copper reducing equivalents (CRE) in the lung tissue of healthy ventilated and non-treated animals (Control group), in non-treated animals with ARDS (ARDS group) and in animals with ARDS treated with olprinone (ARDS/PDE3 group) after the 4h therapy. Statistical comparisons: for ARDS vs. Control ** *p* < 0.01, *** *p* < 0.001; for ARDS/PDE3 vs. ARDS ^#^ *p* < 0.05, ^###^ *p* < 0.001. Data are presented as means ± SEM.

Both observed markers of oxidative damage, 3-nitrotyrosine (3NT) as an indicator of oxidation of proteins (*p* < 0.01), and thiobarbituric acid-reactive substances (TBARS) as an indicator of peroxidation of lipids (*p* < 0.001) were significantly increased in lavage-injured and untreated animals compared to controls (ARDS vs. Control). Total antioxidant capacity (TAC, *p* < 0.001) significantly decreased in ARDS animals compared to controls (ARDS vs. Control). Olprinone therapy decreased levels of both markers of oxidative damage compared to untreated ARDS (3NT, *p* < 0.05; TBARS, *p* < 0.001). On the other hand, TAC significantly increased in the lung tissue of olprinone-treated animals compared to untreated ARDS group (*p* < 0.05) (Figure 2).

### 2.4. Apoptosis in the Lung Tissue

**Figure 3:**
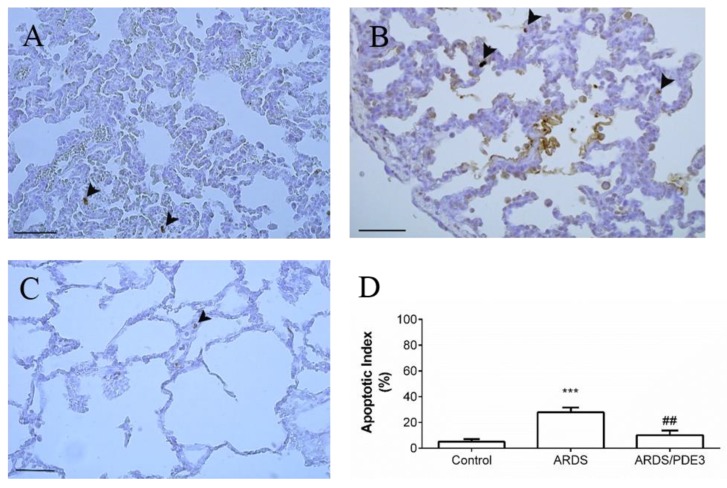
Apoptosis of lung epithelial cells visualized by TUNEL methods after the 4h therapy. Representative microphotographs of the lungs of healthy controls (**A**); animals with non-treated ARDS (**B**) and animals with ARDS treated with olprinone (**C**) using the TUNEL method for detection of apoptosis, scale bars: 50 μm. (**D**) Relative values of the apoptotic index. Arrowheads indicate a higher number of apoptotic (dark brown) nuclei of epithelial cells in the alveoli (A), (B). After treatment with olprinone, a decreased number of apoptotic alveolar cells was found (C). Statistical comparisons: for ARDS vs. Control *** *p* < 0.001; for ARDS/PDE3 vs. ARDS ^##^ *p* < 0.01. Data are presented as means ± SEM.

Apoptotic index (percentage of TUNEL immunoreactive nuclei) was significantly elevated in the saline-lavaged untreated group compared to the control group (ARDS vs. Control, *p* < 0.001), and decreased in olprinone-treated animals (ARDS/PDE3 vs. ARDS, *p* < 0.01; Figure 3).

**Figure 4:**
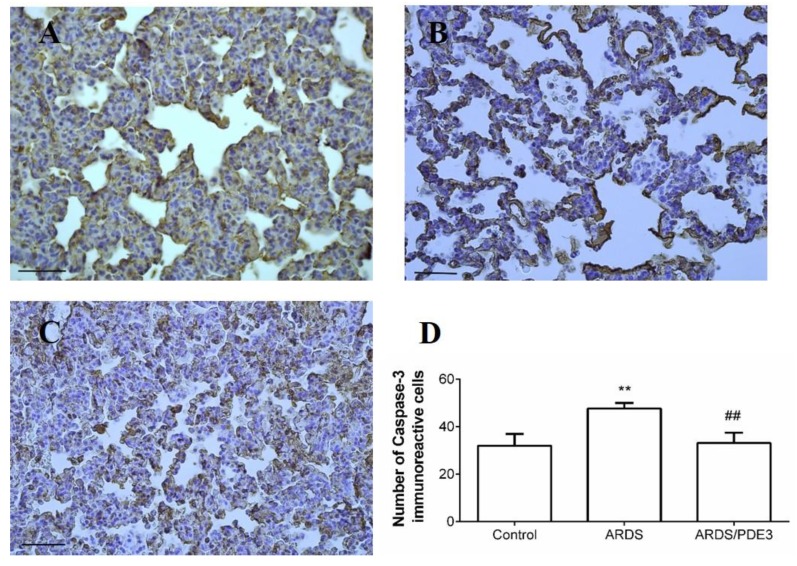
Apoptosis of lung epithelial cells visualized by caspase-3 immunohistochemical staining. after the 4h therapy. Representative microphotographs of the lungs of healthy controls (**A**); animals with non-treated ARDS (**B**) and animals with ARDS treated with olprinone (**C**) using the TUNEL method for detection of apoptosis, scale bars: 50 μm. (**D**) Relative values of the activated caspase-3 immunoreactive alveolar cells. Statistical comparisons: for ARDS vs. Control ** *p* < 0.01; for ARDS/PDE3 vs. ARDS ^##^ *p* < 0.01. Data are presented as means ± SEM.

The number of caspase-3 positive immunoreactive cells in the lung parenchyma increased in the lavage-injured untreated group compared to the control group (ARDS vs. Control, *p* < 0.01). In olprinone-treated animals, the number of caspase-3 immunoreactive cells decreased compared to untreated ARDS animals (*p* < 0.01; Figure 4).

### 2.5. Respiratory Parameters

**Figure 5:**
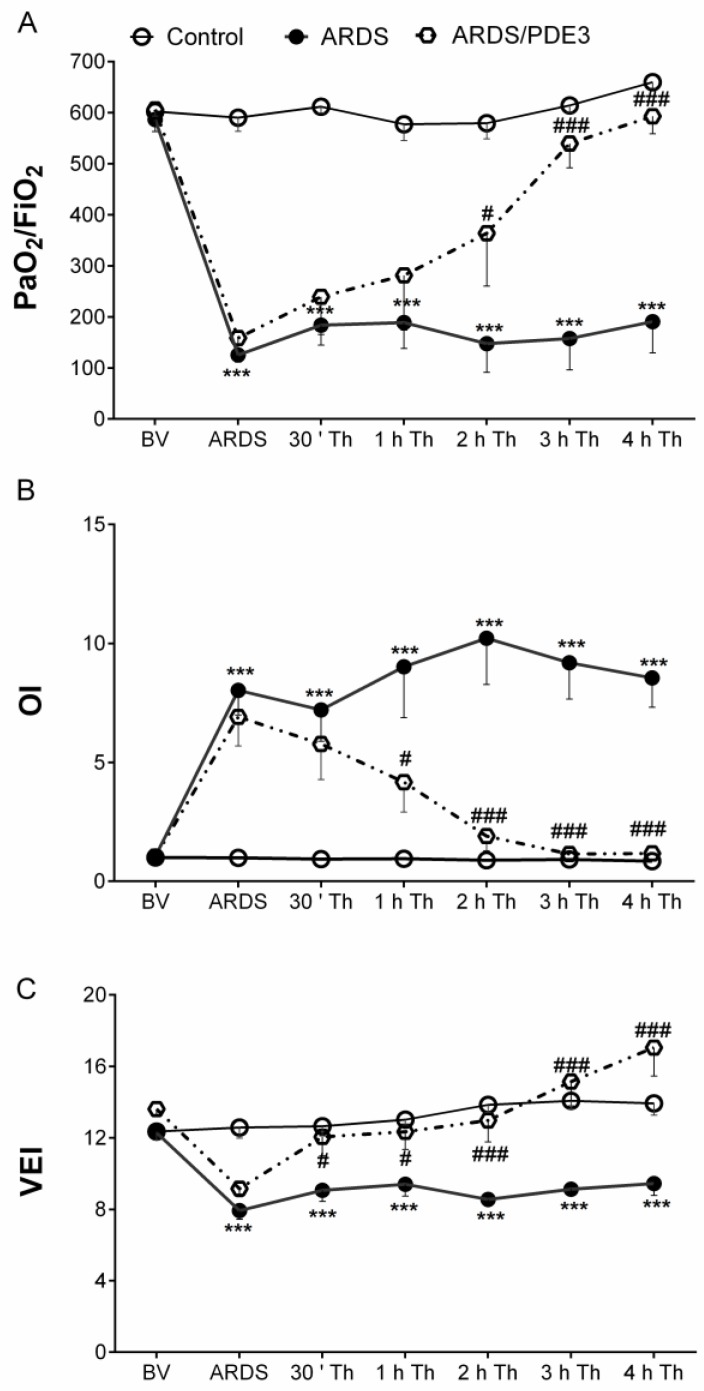
Changes in gas exchange indexes during the experiment. (**A**) The ratio of arterial oxygen partial pressure to the fraction of inspired oxygen (PaO_2_/FiO_2_); (**B**) oxygenation index (OI), and (**C**) ventilation efficiency index (VEI) before (basal value, BV) and after lung lavage (ARDS), 30 min (30′) after ARDS and therapy (Th) administration during 4 h (h) of the experiment in healthy ventilated and non-treated animals (Control group), in non-treated animals with ARDS (ARDS group) and animals with ARDS treated with olprinone (ARDS/PDE3 group). Statistical comparisons: for ARDS vs. Control *** *p* < 0.001; for ARDS/PDE3 vs. ARDS # *p* < 0.05, ### *p* < 0.001. Data are presented as means ± SEM.

Repetitive lung lavage by saline caused a significant worsening in all observed lung function parameters: mean airway pressure (MAP), arterial oxygen saturation (SaO_2_), alveolar-arterial gradient (AAG), dynamic lung–thorax compliance (Cdyn), ratio between partial pressure of oxygen in arterial blood to the fraction of inspired oxygen (PaO_2_/FiO_2_), oxygenation index (OI), and ventilation efficiency index (VEI). Worsening was highly significant when compared to basal values (time sequence ARDS vs. basal value, BV; all *p* < 0.001 for each parameter respectively, in both lavaged groups), but comparable between the lavaged groups. Lavage-induced deterioration of the respiratory parameters persisted in the untreated animals (ARDS group) until the end of the experiment (Table 2 and Figure 5).

Olprinone administration significantly improved Cdyn (*p* < 0.05) and VEI (*p* < 0.05, Figure 5) from 30 min (30’) of therapy (Th), Cdyn (*p* < 0.1), OI (*p* < 0.05), and VEI (*p* < 0.05) from 1 h Th, SaO_2_ (*p* < 0.05), AAG (*p* < 0.01), Cdyn (*p* < 0.01), PaO_2_/FiO_2_ (*p* < 0.05), OI (*p* < 0.001), and VEI (*p* < 0.001) from 2 h Th, MAP (*p* < 0.01), SaO_2_ (*p* < 0.05), AAG (*p* < 0.001), Cdyn (*p* < 0.001), PaO_2_/FiO_2_ (*p* < 0.001), OI (*p* < 0.001), and VEI (*p* < 0.001) from 3 h Th, MAP (*p* < 0.05), SaO_2_ (*p* < 0.001), AAG (*p* < 0.001), Cdyn (*p* < 0.001), PaO_2_/FiO_2_ (*p* < 0.001), OI (*p* < 0.001), and VEI (*p* < 0.001) compared to ARDS group at the end of the experiment (Table 2 and Figure 5). 

## 3. Discussion

Acute respiratory distress syndrome (ARDS) is one of the serious inflammatory diseases that are difficult to cure and/or have a poor prognosis [24]. Pathological features of ARDS include alveolar edema, inflammatory cell infiltration, damage of the lung structure, and worsening of gas exchange [1]. Because the uncontrolled inflammation associated with ARDS often leads to tissue or organ injury and systemic dysfunction [25], the anti-inflammation strategy can be of benefit [26]. In the present study, we have investigated several anti-inflammatory effects of olprinone in the saline lavage-induced model of ARDS. The data showed that olprinone treatment attenuated lung damage and decreased pro-inflammatory cytokines production, oxidative damage, apoptosis, and improved respiratory parameters. 

As recently demonstrated, excessive accumulation of both neutrophils [8] and macrophages [27] contribute to the lung inflammation in ARDS. Macrophages accumulating in the lung early after the injury are activated toward a pro-inflammatory (M1) phenotype, as evidenced by morphologic changes including increased size and vacuolization, and expression of TNFα, prototypical marks of proinflammatory macrophages [27,28,29]. As shown in experimental models, pulmonary damage can be ameliorated or prevented by suppressing or depleting macrophages [30]. When M1 macrophage proinflammatory activity is blocked with anti-inflammatory therapy, lung damage induced by various insults is reduced [31,32]. Increases in M2 macrophages in the lung are correlated with the upregulation of IL-4, IL-10, and IL-13 [33,34]. These cytokines dampen macrophage production of pro-inflammatory mediators and stimulate the generation of extracellular matrix proteins and growth factors important in wound healing [35]. The importance of M2 macrophages in suppressing inflammation and initiating wound repair is associated with increased expression of pro-inflammatory cytokines including TNFα, IL-1β, IL-6, and MCP-1/CCL2 following endotoxin administration, a response correlated with exacerbation of lung inflammation and injury [36].

Repetitive saline lung lavage triggers the migration of polymorphonuclears (PMN), particularly neutrophils, into the alveolar spaces. In our study, higher counts of inflammatory cells were found in the BAL fluid at 4 h after induction of the ARDS model. In agreement with our results, other researchers detected higher PMN count in the BALF after induction of ARDS [37,38,39]. Infiltration of PMN into the airspaces in this study was confirmed by elevated counts of both neutrophils and eosinophils in the BALF at the end of the experiment, similar to other models of ARDS where the increase in both types of PMN was observed [23,40]. These findings suggest that eosinophils participate also in the acute lung inflammation of non-allergic etiology. Although neutrophils and eosinophils do not express PDE3, increasing cAMP levels due to PDE3 inhibition reduces the expression of adhesion molecules and consequently impairs eosinophil and neutrophil adherence to lung endothelial cells [41]. Besides, elevated cAMP levels target p38 MAPK and activate PKA, both leading to inhibition of NF-κB [42,43]. This may explain why olprinone decreased percentages of both neutrophils and eosinophils in BALF in the present study in saline-lavaged ALI as well as in our previous study in a meconium-induced model of ALI [23]. However, the count of macrophages in the BALF increased just slightly which indicates that changes in our experimental model are mediated mostly by PMN.

In ARDS, many pro-inflammatory cytokines participate in the initiation and propagation of the inflammation [44]. Among them, IL-1β and TNFα are the most biologically potent cytokines secreted by activated macrophages in the early phase of ARDS. They stimulate the release of a variety of pro-inflammatory chemokines such as IL-6 and IL-8 with subsequent recruitment of inflammatory cells including neutrophils into the airspaces, alteration of the endothelial–epithelial barrier, and impairment of fluid transport, leading to a generation of alveolar edema [45]. IL-6 as a key inflammatory cytokine strongly promotes the acute inflammatory injury, while its elevated plasma levels correlate with increased mortality rate [46].

In the course of ARDS, the direction and severity of inflammatory response are determined by the degree of interaction between anti-inflammatory and pro-inflammatory factors. When the secretion of anti-inflammatory factors is insufficient, the body cannot resist the inflammatory response, which leads to cell apoptosis exacerbation of the organ dysfunction, and increased mortality rate of patients [47,48,49]. The anti-inflammatory response is supplied by distinct mechanisms including IL-10. The anti-inflammatory cytokine IL-10 is secreted mainly by helper T lymphocytes and macrophages, and effectively restores the balance between anti-inflammatory and pro-inflammatory actions [50]. However, decreased IL-10 is associated with higher mortality in patients with ARDS [51]. In this study, the levels of pro-inflammatory cytokines IL-1β and IL-6 in the lung tissue of ARDS rabbits were significantly increased, but the IL-10 levels were significantly decreased. The mentioned findings confirmed an imbalance between anti-inflammatory and pro-inflammatory responses in rabbits after elicitation of the ARDS model and the occurrence of serious inflammatory response. These results are also consistent with the previous studies [47,48]. In our study, olprinone suppressed the production of pro-inflammatory cytokines in the lung tissues and prevented a decrease in the production of anti-inflammatory cytokine IL-10, which indicated the anti-inflammatory effect of olprinone is at least partially mediated by IL-10 signaling. 

The early phase of ARDS is also associated with injury to epithelial and endothelial lung cells. Receptor for advanced glycation end-products (RAGE) is expressed at low levels in normal lung and becomes upregulated in conditions associated with inflammation and lung damage [52,53,54,55]. RAGE’s inflammatory pathway is not specific for single lung disease, thus, its overexpression has been described, for example, in smoke-related pulmonary disease, organizing pneumonia, granulomatous disease, and usual interstitial pneumonia [53]. High levels of RAGE are constitutively expressed in the alveolar type I (ATI) epithelial cells [56], but in inflammation, the RAGE expression can be also induced in other cell types and tissues [57]. In ATI cells, RAGE is located on the basolateral side [56], enhancing the integrity of the alveolar epithelial layer. RAGE exerts supporting effects on the adhesion of alveolar epithelial cells and spreading on the extracellular matrix [58], impermeability of the alveolar-capillary membrane [59], as well as on the respiratory mechanics [59,60]. RAGE has been recently advocated for one of the specific biomarkers of epithelial injury in ARDS [61], as its increase was observed in BALF and plasma of patients with ARDS [62]. In our study, an increase in RAGE levels was associated with the severity of pulmonary physiological disturbances (PaO_2_/FiO_2_ ratio and compliance), which is consistent with the previous study, where RAGE levels correlated with oxygenation [61].

Inflammation and oxidative stress share overlapping and intersecting pathways. ROS represents both the end-products and inducers of the inflammatory processes [63]. High levels of ROS cause an imbalance of the cellular redox state, oxidative stress, and induce cell apoptosis [64]. In this study, 3-nitrotyrosine (3NT), malondialdehyde (MDA), and total antioxidant capacity (TAC) were chosen as the biomarkers of oxidative damage in the lung tissue. The antioxidant effect of olprinone was indicated by an increase in TAC level and decreases in both MDA and 3NT. The antioxidant properties of PDE inhibitors have been also reported in other studies [23,65,66,67]. Alleviating inflammation and oxidative stress resulted in reduced cellular damage and death. The anti-apoptotic effect of olprinone on the lung epithelial cells was demonstrated by the decrease in the number of caspase-3 positive cells and lower apoptotic index, which was in agreement with recent studies [67,68]. The activation of caspases is one of the intracellular events required for the induction of cell death. Caspases are expressed as inactive zymogens but, upon activation, amplify death stimuli via initiator caspases, caspases 2 and 9 (intrinsic pathway), and caspase 8 and 10 (extrinsic pathway), and lead to dismantling of the cell via executioner caspases 3 and 7 [69]. Irrespective of the initiator exogenous or endogenous cell apoptotic signal pathway, both signaling pathways converge to major executioner caspase-3 [68,70]. Inhibition of caspases, or caspase 3 specifically, attenuates apoptosis and can protect from the lung injury [71,72]. Our results confirmed the role of apoptosis in the pathogenesis of ARDS as demonstrated by increased levels of pro-apoptotic markers. In lavage-injured animals, the number of caspase-3 positive cells increased, while olprinone therapy significantly decreased the number of caspase-3 IR cells in the lung tissue. Increased apoptosis was also shown by another method of apoptosis estimation, TUNEL method, in which the percentage of cells with TUNEL immunoreactive dark brown stained nuclei is determined. This method identifies apoptotic cells early in the apoptotic process before the characteristic morphological changes are obvious [70]. The apoptotic index was also significantly lower in the olprinone-treated group, confirming attenuation of the degree of apoptosis in the lung. Our results are consistent with the recent study, where olprinone treatment inhibited expression of pro-apoptotic proteins Bax and caspase-3, increased expression of anti-apoptotic protein Bcl-2, and elevated the ratio of Bcl-2 to Bax [68].

Acute lung inflammation is associated with increased pulmonary vascular permeability, leading to alveolar flooding and diminished respiratory capacity. In this study, early after the lung lavages the oxygenation and efficacy of ventilation declined and persisted at low levels during the whole experiment. Our current data are consistent with the findings of the earlier studies [73,74] where the saline lavage-induced surfactant-depleted ARDS model was used. Administration of olprinone led to significant improvement of blood gases as well as of measured respiratory parameters and calculated indexes compared to non-treated animals with ARDS, whereas these changes become significant within 1 h after olprinone administration. We presume that the rapid improvement in oxygenation is particularly related to a decrease in the right-to-left pulmonary shunts. Our results are in accordance with previous findings that PDE3 inhibitors can improve oxygenation in various lung injuries, such as meconium aspiration syndrome or pulmonary hypertension in preterm infants [23,75,76]. 

Our study has some potential limitations. First, no single animal model can reproduce all the characteristics of ARDS in humans. The animal model evoked by alveolar lavage with warmed normal saline used in this study is one of the most commonly used ARDS animal models. However, this is primarily a surfactant depletion model, which causes lung injury similar to that in human ARDS, in respect to its effects on oxygenation, pulmonary compliance and atelectasis, and edema, but it induces less macrophage and neutrophil infiltration unless another injury, such as mechanical ventilation, is added [5]. Additionally, the removed surfactant can interfere with lung inflammatory/immune response and oxidative metabolism because the surfactant inhibits neutrophil respiratory activation and has an antioxidant effect on alveolar macrophages [77]. Positive response to olprinone in this animal model widens future perspectives of PDE3 inhibitors also for situations with prominent surfactant depletion and edema formation.

## 4. Materials and Methods

### 4.1. Ethics Statement

The protocol of experiments (Project identification code VEGA 1/0305/14) was approved by the local Ethics Committee of Jessenius Faculty of Medicine, Comenius University, and the National Veterinary Board of Slovak Republic (Ro-3122/13-221 on 23/09/2011). The protocol follows EU Directive 2010/63/EU for animal experiments and complies with the ARRIVE guidelines.

### 4.2. Animals

Adult New Zealand white rabbits (*n* = 21 in total), with body weights (b.w.) of 2.2 to 2.8 kg and approximately 8–11 weeks of age were obtained from Velaz s. r. o. (Prague, Czech Republic). Animals were housed one or two per cage in transparent cages with a plastic bottom grid without bedding, provided by an elevated resting platform and enhanced by wooden blocks, at a temperature of 20 °C and 45–60% humidity, and under 12/12 h light/dark cycle. Rabbits were fed by standard diet (Velaz s. r. o.) once per day according to the weight range and had access to water *ad libitum*.

### 4.3. The General Design of Experiments

**Figure 6:**
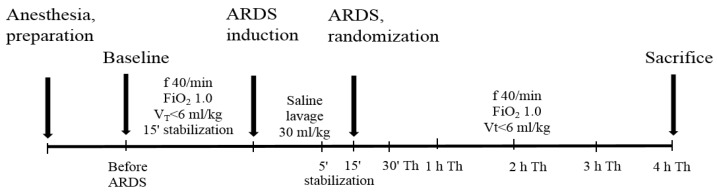
Timeline representation of the experimental protocol. ARDS = acute respiratory distress syndrome, V_T_ = tidal volume, f = frequency, FiO_2_ = fraction of inspired oxygen.

After anesthetic and surgical procedures described earlier [78], animals were subjected to ventilator Aura V (Chirana, Slovakia) and were ventilated conventionally with following settings: frequency (f) of 40/min, the fraction of inspired oxygen (FiO_2_) of 1.0, time of the inspiration (Ti) 50%, and tidal volume (V_T_) < 6 mL/kg b.w. After 15 min (15’) of stabilization, arterial blood samples were obtained for analysis of blood gases (RapidLab 348, Siemens, Germany). One group of healthy ventilated animals served as controls (Control group, *n* = 7). In other animals, lung injury was induced by a repetitive lung lavage with 0.9% saline (30 mL/kg b.w., 37 °C) which was instilled into the endotracheal cannula in the semi-upright right and left lateral positions of the animal and was immediately suctioned by a suction device. Lavage was performed 6–12 times until PaO_2_ decreased to < 26.7 kPa in FiO_2_ 1.0 in two measurements at 5 min (5’) and 15 min (15’) after the lavage. When the criteria for the ARDS model were full-filled, animals were treated with olprinone hydrochloride, dissolved in saline at the dose of 1 mg/kg b.w. (ARDS/PDE3 group, *n* = 7; Sigma-Aldrich, St. Louis, MO, USA), which was given intravenously for 2 min, or were left without therapy (ARDS group, *n* = 7). All animals were oxygen-ventilated (FiO_2_ 1.0, frequency 40/min, V_T_ < 6 mL/kg b.w.) for an additional 4 h after administration of the treatment. At the end of the experiment, animals were overdosed by anesthetics (Figure 6).

### 4.4. Analysis of Cells in the Bronchoalveolar Lavage Fluid (BALF) 

After sacrificing the animal, lungs and trachea were excised. The left lung was lavaged with saline (0.9% NaCl, 37 °C, 3 × 10 mL/kg b.w.). The total numbers of cells in the BALF were determined by automated cell counter Countess^TM^ (Invitrogen, Waltham, MA, USA) and were expressed in absolute values (×10^3^/mL). Then, the BALF was centrifuged 1500 rpm for 15 min. Differential counts of cells in the BALF sediment were evaluated microscopically after staining by May-Grünwald/Giemsa-Romanowski and were expressed in absolute values (×10^3^/mL).

### 4.5. Detection of Markers of Inflammation and Oxidative Damage

Concentrations of cytokines and oxidative modification products were determined in 10% (weight/volume) lung homogenate prepared using 0.1 M ice-cold phosphate buffer (PBS, pH 7.4) by Polytron homogenizer PT 1200 E (5-times for 25 s, 1200 rpm; Kinematica AG, Switzerland). Homogenates were then frozen 3 times and centrifuged (12,000 rpm, 15 min, 4 °C). Final supernatants were then stored at −70 °C until the analysis was performed.

Concentrations of IL-6, IL-1β, IL-10 (USCN Life Science Inc., Wuhan, China), and RAGE (MyBioSource, San Diego, CA, USA) were quantified using rabbit-specific ELISA kits according to the manufacturers’ instructions. Data were expressed in pg/mL.

Protein oxidative damage was determined using OxiSelect^TM^ Nitrotyrosine ELISA Kit (Cell Biolabs, Inc., San Diego, CA, USA). Data were expressed in 3-nitrotyrosine nanomole concentration (nM, 3NT). Lipid oxidative damage expressed by the concentration of thiobarbituric acid reacting substances (TBARS) was determined by OxiSelect^TM^ TBARS Assay Kit (Cell Biolabs, Inc., San Diego, CA, USA). Data were expressed as malondialdehyde in micromole concentration (μM MDA).

Total antioxidant capacity (TAC) was determined using an ELISA kit according to the manufacturer’s instructions (Cell Biolabs, Inc., San Diego, CA, USA). Data were expressed as micromole concentration of copper reducing equivalents (μM CRE). 

### 4.6. Lung Epithelial Cells Apoptosis Assays

#### 4.6.1. In Situ Labeling of DNA Strand Breaks by TUNEL Method

The lungs were immersed in 4% formalin solution. After paraffin embedding, the 4 µm thick slides were cut on microtome followed by deparaffinization and pretreatment with a proteinase K. The tissue sections were further processed by DeadEnd^TM^ Colorimetric TUNEL System (Promega, Durham, NC, USA). This assay labels the fragmented DNA of apoptotic cells. The biotinylated nucleotide is incorporated at the 3’-OH DNA ends using the Terminal Deoxynucleotidyl Transferase, Recombinant, (rTdT) enzyme. Horseradish peroxidase-labeled streptavidin (Streptavidin HRP) is then bound to these biotinylated nucleotides. For the detection of nucleotides and blocking endogenous peroxidases, the sections were incubated with 0.3% H_2_O_2_ solution. The colour of sections was developed after incubation with diaminobenzidine, DAB-chromogen solution. The sections were then counterstained with Mayer’s hematoxylin and mounted with a Permount (Fisher, Waltham, MA, USA). The slides were viewed with an Olympus BX41 microscope (Olympus, Japan). The image capture was performed with Quick Photo Micro software, version 2.2 (Olympus). The apoptotic index was calculated as the percentage of TUNEL immunoreactive (TUNEL-IR) dark brown stained nuclei in a total of 100 nuclei randomly counted from three sites within each section.

#### 4.6.2. Immunohistochemistry for Activated Caspase 3 

After deparaffinization, revitalization, and rehydration, the tissue slides were treated with a 3% H_2_O_2_ solution for 10 min for blocking endogenous peroxidases. Washing with Tris buffer was used after each handling step. The sections were incubated with the primary antibody rabbit anti-caspase 3 (1:500; Bioss, Woburn, MA, USA) for 30 min at room temperature. The specimen was then incubated by sequential 10 min. incubation with biotinylated anti-rabbit secondary antibody and peroxidase-labeled streptavidin conjugated to HRP (DAKO LSAB^®^2 System-HRP; Dako, Denmark). The colour of the sections was developed after incubation with the DAB-chromogen solution (Dako). The sections were then counterstained with Mayer’s hematoxylin and mounted with Entellan (Merck, Kenilworth, NJ, USA). The slides were viewed with an Olympus BX41 microscope (Olympus, Japan). The image capture was performed with Quick Photo Micro software, version 2.2 (Olympus). The density of activated caspase-3 immunoreactive cells (dark-brown cytoplasm; caspase 3-IR) was measured randomly from three sites within each section and was calculated as the total number of caspase 3-IR cells in the field. 

### 4.7. Measurement of Respiratory Parameters

During the experiment, animals were ventilated with ventilator Aura V (Chirana, Slovakia). Ventilatory parameters, such as FiO_2_, V_T_, minute ventilation, f, Ti, ventilatory pressures (mean airway pressure (MAP), PIP and PEEP), dynamic (Cdyn), and static (Cst) lung compliance, and airway resistance (Raw) were automatically measured by in-build sensors and software and were displayed on the screen of the ventilator. Partial pressures of oxygen and carbon dioxide (PaO_2_ and PaCO_2_), arterial oxygen saturation (SatO_2_), and parameters of acid-base balance were measured with a blood gas analyzer (Rapidlab 348, Siemens, Germany). Ventilation efficiency index was calculated as VEI = 3800/((PIP-PEEP) × frequency × PaCO_2_). The oxygenation index (OI) was calculated as: OI = (MAP (kPa) × FiO_2_ (%))/PaO_2_ (kPa). The PaO_2_/FiO_2_ ratio was calculated as: PaO_2_/FiO_2_ = (PaO_2_ (mmHg) × 100)/FiO_2_ (%). Alveolar partial pressure of O_2_ (P_A_O_2_ mmHg) was expressed as: P_A_O_2_ = {(FiO_2_ (%)/100) × (Patm − P_H2O_)} – (PaCO_2_ (mmHg)/RQ), whereas atmospheric pressure (Patm) at a sea level is 760 mmHg, P_H2O_ is 47 mmHg, and RQ (respiratory quotient) is 0.8. Alveolar–arterial gradient (AAG) was calculated as: AAG = P_A_O_2_ (mmHg) − PaO_2_ (mmHg).

### 4.8. Statistical Analyses of Results

GraphPad Prism (Version 6.01) (GraphPad Software, San Diego, California, USA) software was used for statistical analyses. Statistical differences between the groups were determined by analysis of variance (ANOVA) with Bonferroni post-hoc test or Kruskal-Wallis test. A P value below 0.05 was considered to be statistically significant. Results are presented as average with error bars indicating standard error of the mean (means ± SEM).

## 5. Conclusions

This study demonstrated that in the saline lavage-induced model of ARDS the PDE3 inhibitor olprinone protected from the acute lung injury. Favorable effects of olprinone may be related to its antioxidant properties, but they may be also related to the suppression of the aggravated inflammation and lung epithelial cell damage. The results suggest that the administration of olprinone may represent a novel treatment for inflammatory lung damage. 

## Figures and Tables

**Table 1:** Total count and differential leukocyte count (both expressed in absolute value ×10^3^/mL) in the bronchoalveolar lavage fluid (BALF) before (basal value, BV) and in the 4 h of the therapy (Th) in healthy ventilated controls (Control), untreated group with acute respiratory distress syndrome (ARDS), and ARDS group treated with phosphodiesterase-3 (PDE3) inhibitor olprinone (ARDS/PDE3). Data are presented as means ± SEM. Ma–macrophages, Neu–neutrophils, Eos–eosinophils. Statistical comparisons: for ARDS vs. Control ** *p* < 0.01, *** *p* < 0.001; for ARDS/PDE3 vs. ARDS ^#^ *p* < 0.05, ^###^ *p* < 0.001. Data are presented as means ± SEM.

**Total Count (×10^3^/mL)**				
		Control	ARDS	ARDS/PDE3
	BV	157.5 ± 49.5	194.4 ± 45.7	196.6 ± 54.8
	4h Th	250.0 ± 48.2	1358.8 ± 380 **	503.3 ± 174.0 ^#^
**Differential Count (×10^3^/mL)**				
Ma	BV	155.8 ± 49.0	190.8 ± 38.2	189.5 ± 54.3
	4h Th	240.1 ± 50.5	229.8 ± 56.4	184.6 ± 56.6
Neu	BV	1.4 ± 0.5	2.9 ± 0.6	5.9 ± 2.8
	4h Th	7.6 ± 2.2	1098.8 ± 316.6 ***	312.6 ± 127.3 ^###^
Eos	BV	0.3 ± 0.2	0.6 ± 0.2	1.2 ± 0.6
	4h Th	2.3 ± 0.7	30.1 ± 6.5	6.1 ± 1.9

**Table 2:** Mean airway pressure (MAP), arterial oxygen saturation (SaO_2_), alveolar–arterial gradient (AAG), and dynamic lung–thorax compliance (Cdyn) before (basal value, BV) and after lung lavage (ARDS) and within 4 h after administration of the therapy (Th) in healthy ventilated and non-treated animals (Control group), in non-treated animals with ARDS (ARDS group) and in animals with ARDS treated with olprinone (ARDS/PDE3 group). Statistical comparisons: for ARDS vs. Control * *p* < 0.05, ** *p* < 0.01, *** *p* < 0.001; for ARDS/PDE3 vs. ARDS ^#^ *p* < 0.05, ^##^ *p* < 0.01, ^###^ *p* < 0.001. Data are presented as means ± SEM.

	Before ARDS	After ARDS	0.5 h Th	1 h Th	2 h Th	3 h Th	4 h Th
**MAP (kPa)**							
Control	0.8 ± 0.0	0.8 ± 0.0	0.8 ± 0.0	0.8 ± 0.0	0.7 ± 0.0	0.7 ± 0.0	0.7 ± 0.0
ARDS	0.8 ± 0.0	1.1 ± 0.0 ***	1.0 ± 0.0 ***	1.0 ± 0.0 ***	1.0 ± 0.1 ***	1.0 ± 0.1 ***	1.1 ± 0.1 ***
ARDS/PDE3	0.8 ± 0.0	1.0 ± 0.0	1.0 ± 0.0	1.0 ± 0.1	0.9 ± 0.1	0.8 ± 0.0 ^##^	0.9 ± 0.0 ^#^
**SaO_2_**							
Control	99.9 ± 0.0	99.9 ± 0.0	99.9 ± 0.0	99.9 ± 0.0	99.9 ± 0.0	99.9 ± 0.0	99.9 ± 0.0
ARDS	99.9 ± 0.0	90.0 ± 2.1 **	89.9 ± 2.4 *	87.4 ± 2.6 ***	86.9 ± 2.3 ***	86.8 ± 2.5 ***	82.2 ± 3.9 ***
ARDS/PDE3	99.9 ± 0.0	93.4 ± 2.7	95.8 ± 1.3	95.4 ± 1.6	97.4 ± 1.1 ^#^	97.3 ± 1.8 ^#^	98.5 ± 1.0 ^###^
**AAG**							
Control	612.0 ± 11.7	593.9 ± 33.6	606.6 ± 14.7	561.9 ± 41.1	563.3 ± 38.0	601.9 ± 13.5	663.2 ± 14.6
ARDS	546.8 ± 25.3	121.9 ± 15.1 ***	144.8 ± 33.1 ***	130.0 ± 38.9 ***	144.0 ± 52.6 ***	148 ± 51.8 ***	139.6 ± 38.1 ***
ARDS/PDE3	606.2 ± 10.3	169.9 ± 37.5	239.5 ± 74.1	281.5 ± 107.3	363.8 ± 103.4#	540 ± 48 ^###^	593.0 ± 35 ^###^
**Cdyn (mL/kPa)**							
Control	14.5 ± 0.54	14.7 ± 0.4	14.7 ± 0.5	15.2 ± 0.4	15.0 ± 0.4	14.8 ± 0.3	14.9 ± 0.5
ARDS	13.0 ± 0.8	6.9 ± 0.4 ***	7.9 ± 0.4 ***	7.5 ± 0.5 ***	7.3 ± 0.4 ***	6.2 ± 0.9 ***	6.8 ± 0.4 ***
ARDS/PDE3	14.1 ± 0.5	8.3 ± 0.4	9.9 ± 1.0 ^#^	9.9 ± 0.5 ^##^	10.0 ± 0.4 ^##^	9.3 ± 0.8 ^###^	10.2 ± 0.3 ^###^
